# Privacy-Preserving Energy Management of a Shared Energy Storage System for Smart Buildings: A Federated Deep Reinforcement Learning Approach

**DOI:** 10.3390/s21144898

**Published:** 2021-07-19

**Authors:** Sangyoon Lee, Le Xie, Dae-Hyun Choi

**Affiliations:** 1 School of Electrical and Electronics Engineering, Chung-Ang University, 84 Heukseok-ro, Dongjak-gu, Seoul 156-756, Korea; sangyoon1207@naver.com; 2 Department of Electrical and Computer Engineering, Texas A&M University, College Station, TX 77843, USA; le.xie@tamu.edu

**Keywords:** building energy management system, shared energy storage system, federated reinforcement learning, deep reinforcement learning, smart buildings

## Abstract

This paper proposes a privacy-preserving energy management of a shared energy storage system (SESS) for multiple smart buildings using federated reinforcement learning (FRL). To preserve the privacy of energy scheduling of buildings connected to the SESS, we present a distributed deep reinforcement learning (DRL) framework using the FRL method, which consists of a global server (GS) and local building energy management systems (LBEMSs). In the framework, the LBEMS DRL agents share only a randomly selected part of their trained neural network for energy consumption models with the GS without consumer’s energy consumption data. Using the shared models, the GS executes two processes: (i) construction and broadcast of a global model of energy consumption to the LBEMS agents for retraining their local models and (ii) training of the SESS DRL agent’s energy charging and discharging from and to the utility and buildings. Simulation studies are conducted using one SESS and three smart buildings with solar photovoltaic systems. The results demonstrate that the proposed approach can schedule the charging and discharging of the SESS and an optimal energy consumption of heating, ventilation, and air conditioning systems in smart buildings under heterogeneous building environments while preserving the privacy of buildings’ energy consumption.

## 1. Introduction

A shared energy storage system (SESS) is a promising technology to efficiently manage the energy consumption in residential and commercial sectors. Compared to individual ESSs, installment of the SESSs reduces the installation costs for individual consumers and minimizes the operation costs of ESS [1]. Recently, smart homes and buildings are equipped with advanced smart grid technologies, including distributed energy resources (DERs) such as a solar photovoltaic (PV) system and Internet of Things-based home/building appliances along with advanced energy sensors, namely smart meters, that monitor the real-time energy usage of consumer through advanced metering infrastructure network. The advancement of these smart grid technologies combined with the SESS suggests the need for more intelligent energy management systems that save electricity cost for consumers while maintaining their comfort levels and preferences.

Conventional algorithms for the energy management of buildings connected to the SESS have been developed using various model-based optimization methods. However, the model-based approach is formulated with approximated constraints for the operation characteristics of appliances so that it may yield an inaccurate and diverging solution. To address the limitation, this study presents a machine learning (ML) method for developing the energy management framework for a SESS with multiple smart commercial buildings.

Building energy management systems (BEMSs) are the core solutions for BEMS operators to monitor the real-time building energy usage through smart meters and accordingly, manage the optimal energy consumption of building appliances (e.g., heating, ventilation, and air conditioning (HVAC) systems and lights), thereby reducing the electricity cost while ensuring the comfort and satisfaction of the building’s occupants. Approximately 45% of the total electricity consumption in the building comes from the HVAC system; therefore, the primary goal of the BEMS is to calculate the optimal energy consumption schedule of the HVAC system and consequently, reduce the electricity cost of the building.

There have been various optimization methods (e.g., robust optimization, stochastic programming, and model predictive control (MPC)) for the energy management of smart residential and commercial buildings while considering their uncertain and dynamic operation characteristics. For the robust optimization method, the range of the values of the uncertain parameters is determined by their continuous set. By contrast, for the stochastic programming method, the uncertain parameters are characterized by a number of discrete probabilistic scenarios. MPC is an optimization control approach that predicts the dynamic system operation characteristics over a predefined horizon time period for minimizing the cost function of the system via optimal control decisions. A robust optimization-based BEMS for a smart building with a wind turbine was developed in [2] where the total cost of heat/power co-generation and the total cost of emissions are minimized while considering the uncertainty of the wind speed and electrical and thermal loads. In [3], a robust optimization algorithm for the energy management of smart home with PV system, ESS, and electric vehicle (EV) was presented. This algorithm consisted of two stages where the exact energy consumption schedule for smart home is determined using a deterministic optimization method at the first stage and a heuristic algorithm is executed to reflect the uncertainty of the power production and energy demand for extended simulation time horizon. In [4], a stochastic programming was employed to construct home energy management system where the uncertainty of DERs and the EV availability for charging/discharging are incorporated in the scheduling of home energy consumption. In [5], a stochastic optimization algorithm was presented to minimize the energy cost and emissions of residential microgrids. In this algorithm, a scenario-based approach was employed to reflect the uncertainties of electrical market price, electrical and thermal load, and PV generation output. In [6], a BEMS method using the MPC method was presented where the energy consumption of commercial building is reduced by optimizing the operation of HVAC system in 24 h prediction horizon. In [7], a stochastic MPC strategy for the energy management of a smart home with DERs was formulated and executed in a real-time operation mode where the prediction horizon is set to 24 h.

Recently, considerable effort has been devoted to developing the optimization-based BEMS algorithms considering the operation of HVAC system. HVAC control methods for minimizing the electricity cost of commercial and university buildings were presented in [8,9]. In [10], an HVAC management algorithm that considers random occupancy in buildings was proposed. The BEMS method that involves multiple environmental variables of buildings, such as indoor temperature and lighting, was developed under a time-of-use (TOU) price-based demand response program in [11]. Currently, smart communities with various DERs are emerging; therefore, the SESS control algorithms have been developed as a core technology for efficient and economic energy management for multiple residential and commercial buildings. These algorithms range from the scheduling of the SESS for commercial building energy management under demand side management [12], the development of a novel cooperative algorithm between the SESS and residential buildings [13], credit-based operation of the SESS among households using Lyapunov optimization method [14], the scheduling of the SESS operation with a shared PV system based on welfare optimization and a game theoretical method according to consumers’ preferences [15], and the impact analysis of the SESS operation subject to data privacy [16].

Note that the aforementioned optimization-based BEMS methods with the SESS highly rely on abstract optimization models and have a limited capability in larger system models with uncertain input data, thereby leading to a significantly high computational complexity and in some cases, even yielding a diverging solution. To handle these limitations from the model-based BEMS with the SESS, deep reinforcement learning (DRL) has recently attracted attention for being a model-free BEMS approach. A DRL algorithm using deep Q-network and deep policy gradient was adopted to classify the patterns of building energy usage and optimize the energy consumption schedule of the building in [17]. In [18], a joint DRL approach for datacenter and HVAC load control was proposed to reduce the total energy cost within the range of the preferred room temperature while satisfying datacenter workload deadline constraints. In [19], an imitation learning approach was employed to the DRL-based HVAC control algorithm to identify the characteristic of HVAC system controller in the building. A multi-agent DRL framework was developed in [20] where the energy cost of HVAC system in a multi-zone commercial building is minimized while considering random zone occupancy, consumer’ thermal comfort, and air quality comfort. A concise review of DRL-based smart building energy management methods is summarized in [21]. More recently, various DRL-based BEMS methods have been developed, including the scheduling of the ESS and HVAC in residential buildings based on a deep deterministic policy gradient (DDPG) method [22], the management of utility-scale interruptible loads in a dueling deep Q network [23], actor–critic-based demand response management for industrial facilities [24], and the control of the state of charge of a group of multiple ESSs using the DDPG method [25].

However, in the aforementioned centralized DRL-based frameworks, the consumer’s private data aggregated at a global server (GS) can be exposed to attackers who may exploit these data for finding the energy consumption behavior of consumers. There has been many studies to present energy management methods while considering the privacy of consumers in the residential and commercial buildings [26,27,28,29]. In [26], a Kullback–Leiber divergence was adopted to assess the privacy leakage in smart meters installed at the residential buildings and the energy management strategy was formulated with a weighted sum of the consumer privacy and energy cost. A service architecture of home energy management system for preserving the privacy of smart meter data was designed in [27] where the abuse of smart meter data by an adversary can be readily identified. In [28], a new privacy-preserving method for households with the ESSs was presented where an energy usage manipulation by an adversary can be prevented by using the ESS opportunistically. In [29], a privacy-enhanced architecture for the commercial buildings was proposed where the occupancy data are distorted to hide individual occupant location information while ensuring the performance of HVAC. Recently, federated learning (FL) [30] was proposed to preserve the consumer privacy. It is an ML method employed for training local agents and building their optimal models in a distributed manner. In FL, local and global neural network models between the local agents and the GS are exchanged without their private local data being shared to preserve consumer data privacy. Furthermore, for applying this FL concept to various engineering control problems, federated reinforcement learning (FRL) [31] was proposed wherein the optimal policy for individual agent was calculated as long as ensuring that the data were not shared among agents during the training process. The FRL method was employed for developing the navigation for cloud-based robotic systems [32], rapid personalization process of agents [33], and as a defense strategy for jamming attacks in the flying ad hoc network for unmanned aerial vehicles [34]. FRL has been adopted to resolve distributed control problems in a variety of engineering fields. Our recent study [35] developed a privacy-preserving FRL framework to manage the energy consumption of multiple smart homes with DERs. In this study, the DRL agents for home appliances such as air conditioners, washing machines and residential ESS iteratively interact with the GS to build their optimal energy consumption model in multiple homes.

However, to the best of the authors’ knowledge, no study has been performed to construct a privacy-preserving DRL-based SESS model with an FRL-based BEMS model to manage the energy scheduling of the SESS and energy consumption of the HVACs in buildings in a distributed manner. In comparison with our previous study [35] where no SESS was considered in the FRL-based home energy management system, the proposed approach has applied the FRL method to multiple commercial buildings integrated with the SESS. In the previous study, the role of the GS was limited to only aggregating the local agents’ models and broadcasting it back to the local agents without conducting the DRL training procedure. On the other hand, in the proposed approach, the GS is designed to train the charging and discharging schedule of the SESS through the DRL process as well as update the local models. Furthermore, different from the previous study, the proposed approach can further enhance the privacy-preserving of local agents in the FRL framework by adopting a selective parameter method [36] in which all local agents choose a randomly chosen part of their local model and deliver it to the GS. Lastly, a novel method for maintaining stable charging/discharging scheduling of the SESS was presented while preserving the private energy data of buildings.

**Figure 1:**
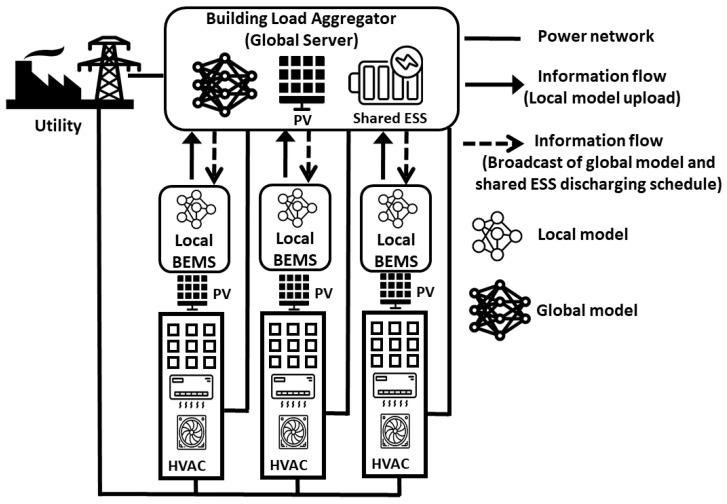
System architecture of a federated reinforcement learning (FRL)-based building energy management system (BEMS) with a shared energy storage system (SESS).

Figure 1 shows a system model of the proposed FRL-based BEMS with a SESS. In this model, we considered three entities: (i) an electric utility, (ii) a local BEMS (LBEMS) operator, and (iii) a non-profit building load aggregator (BLA) with the GS. The GS interacts with the LBEMSs to build the optimal energy consumption model of the HVACs and the optimal charging and discharging model of the SESS by performing the following two tasks: (i) the FRL process through an exchange of local neural network models and global neural network model for the energy consumption of the HVAC between the GS and LBEMSs, and (ii) SESS energy charging from the grid (i.e., energy purchase from the BLA) or energy discharging to the LBEMSs by the GS.

The main contributions of this paper are provided as follows:

- We present a distributed FRL architecture in which the energy consumption of smart buildings and the energy charging and discharging of the SESS are optimally scheduled within the heterogeneous environments of the buildings while preserving the privacy of energy usage information of individual buildings.
- We develop a robust privacy-preserving FRL-based BEMS algorithm against building privacy leakage in the hierarchically distributed architecture. During the FRL process, the HVAC DRL agent improves the energy consumption model of the LBEMS through an iterative interaction with the GS and preserves the privacy of energy usage data using a selective parameter sharing method. Subsequently, the SESS DRL agent trains the optimal energy charging and discharging model of the SESS by using the LBEMS agent’s neural network model without sharing the relevant energy consumption data to preserve the privacy of the buildings’ energy consumption.

The rest of the paper is organized as follows. Section 2 introduces the DRL and FRL methods in the Markov Decision Process (MDP). The mathematical formulation and methodology for the proposed approach are developed in Section 3. Section 4 provides the simulation results for the proposed algorithm with the SESS under heterogeneous building environments. Finally, discussions and concluding remarks are drawn in Section 5 and Section 6, respectively.

## 2. Background of Reinforcement Learning

### 2.1. Markov Decision Process (MDP)

An MDP is defined as a 5-tuple (S,A,P,R,T) in which S is a finite set of all valid states and A is a finite set of all valid actions. The function P:S×A→P(S) represents a Markovian transition model that is the transition probability function, where $P(s_{t+1}|s_{t},a_{t})$ denotes the probability of transitioning from state $s_{t}$ at time *t* into state $s_{t+1}$ at time t+1 after an agent selects an action $a_{t}$ in state $s_{t}$ at time *t*. R:S×A×S→R is the reward function such that a reward $R_{t+1}=R\left(s_{t},a_{t},s_{t+1}\right)$ is obtained from transitioning from state $s_{t}$ into state $s_{t+1}$ after taking an action $a_{t}$. To solve an MDP problem having a finite time horizon *T* equals to determine a policy $\pi_{\theta }\in \Pi$ in which π is parameterized with θ that are the weights and biases of a neural network. The policy $\pi_{\theta }$ finds the action a∈A that should be conducted in any state *s* in order to maximize the discounted cumulative rewards that an agent receives from the state-action transition procedure during a finite time horizon *T*. In a Q-learning method, the policy $\pi_{\theta }$ relies on the value of $Q(s_{t},a_{t})$, namely the Q-value, which estimates the propriety of selected action $a_{t}$ in given state $s_{t}$. This Q-value is estimated for the sum of the discounted cumulative future rewards and can be expressed as $Q\left(s_{t},a_{t}\right)=E\left[\sum_{i=0}^{t}\gamma^{i}R_{t+1+i}|s=s_{t},a=a_{t}\right]$. γ∈[0,1] is a discounting factor, and it represents the relative importance of present and upcoming rewards. The Q-learning method aims to find the optimal policy $\pi_{\theta }^{*}$ that maximizes the Q-value (i.e., $\pi_{\theta }^{*}=\arg\max_{\pi_{\theta }}Q\left(s_{t},a_{t}\right)$) when the agent executes the action according to policy $\pi_{\theta }$.

### 2.2. Deep Reinforcement Learning

An actor–critic algorithm is a contemporary DRL method that uses both policy-based DRL and value-based DRL [37]. In this DRL method, the agent applies two different types of networks to evaluate the goodness of the selected action in given state. The policy network which selects the action and returns its probability is actor network, and the network that estimates the value of the agent’s given state is the critic network. The policy gradient method is suitable for handling the problem with a continuous action space; however, this method may cause a poor convergence performance. An additional critic network in the actor–critic algorithm can improve this convergence issue from the policy gradient method. The objective of the actor–critic algorithm is to proceed its learning to minimize the sum of two different loss functions, which correspond to $L_{t}^{\mathrm{actor}}\left(\theta_{a}\right)$ and $L_{t}^{\mathrm{critic}}\left(\theta_{c}\right)$ for the actor network and critic network, respectively:

(1) $$\begin{array}{c} \min L_{t}^{\mathrm{actor}}\left(\theta_{a}\right)+L_{t}^{\mathrm{critic}}\left(\theta_{c}\right) \end{array}$$

s.t.

(2) $$\begin{array}{cc} L_{t}^{\mathrm{actor}}\left(\theta_{a}\right) & =-\log p_{\pi_{\theta_{a}}}\left(a_{t}|s_{t}\right)Q_{\pi_{\theta_{c}}}\left(s_{t},a_{t}\right) \end{array}$$

(3) $$\begin{array}{cc} L_{t}^{\mathrm{critic}}\left(\theta_{c}\right) & ={\left(TD_{t}-V_{\pi_{\theta_{c}}}\left(s_{t}\right)\right)}^{2}. \end{array}$$

In Equation (1), $\theta_{a}$ and $\theta_{c}$ represent the parameters of the actor network and the critic network, respectively. In Equation (2), $p_{\pi_{\theta_{a}}}\left(a_{t}|s_{t}\right)$ implies the probability of selecting action $a_{t}$ given state $s_{t}$ at time *t* under policy $\pi_{\theta_{a}}$. Moreover, $Q_{\pi_{\theta_{c}}}\left(s_{t},a_{t}\right)$ represents the Q-value of the agent’s action $a_{t}$ at state $s_{t}$ under policy $\pi_{\theta_{c}}$. In Equation (3), the target value of the critic network is denoted by $TD_{t}=R_{t+1}+\gamma V_{\pi_{\theta_{c}}}\left(s_{t+1}\right)$. This target value can be calculated using a temporal difference (TD) method [38] for updating the critic network. $V_{\pi_{\theta_{c}}}\left(s_{t}\right)$ represents the value of state $s_{t}$ under policy $\pi_{\theta_{c}}$. $V_{\pi_{\theta_{c}}}\left(s_{t}\right)$ is written as the expectation of the discounted cumulative future reward that the agent will obtain in state $s_{t}$ as follows: $V_{\pi_{\theta_{c}}}\left(s_{t}\right)=E\left[R_{t+1}+\gamma R_{t+2}+\gamma^{2}R_{t+3}+\cdots |s=s_{t}\right]$.

### 2.3. Federated Reinforcement Learning

As mentioned earlier, FRL is one of the prospective ML methods to train the agents for different local devices in a distributed manner without allowing all agents to share their data for preserving their privacy. The learning process for FRL consists of the following two steps: (i) training for local models and (ii) assembling and updating local models with a newly generated global model. Assume that N:={1,2,…N} is a set of *N* local agents. Each agent *n* constructs its own optimal neural network model $\omega_{n}$ through its training process using its own dataset $D_{n\in N}$. After finishing the training procedure of each local agent, each agent *n* transmits its model $\omega_{n}$ to a GS. Subsequently, the GS aggregates all local models to its batch $\varphi =[\omega_{1},\omega_{2},\ldots ,\omega_{N}]$ and builds the global model $\omega_{G}$ using the batch by $\omega_{G}=f\left(\varphi \right)$. Further, the global model $\omega_{G}$ is broadcasted to all connected local agents and their models are updated by $\omega_{G}$: $\omega_{G}=\omega_{1}=\omega_{2}\ldots =\omega_{N}$. Finally, each agent restarts its training using an identical $\omega_{G}$ along with dataset $D_{n\in N}$ during the iterative communication round. This iterative interaction between all local agents and the GS continues until all local agents find their optimal model. In a centralized model for existing DRL methods, an agent in the GS must gather the data of all local devices at a single location to construct the neural network models for all local devices. This centralized approach may result in data privacy leakage from local devices. In addition, large volume of data collected at the GS may increase the training time of the agent significantly. However, in the FRL approach, no local data sharing is required in the GS. Consequently, FRL can successfully preserve the privacy of local data for agents. In addition, since FRL trains the agents for different local devices in a distributed manner, it can reduce the training time of the agents significantly. By contrast, a distributed multi-agent model without the GS may reduce the training time because each agent trains only its model by using its own data. However, in this model, some agent may not have enough data, and it results in an overfitting problem (i.e., the trained local model becomes biased, thereby leading to inaccurate policy). Note that during the FRL process the local models of devices are periodically updated by the global model that is constructed by the GS. The periodical local model update by the global model can improve the local models, thereby preventing the overfitting issue of local models.

## 3. Energy Management of a Shared ESS for Smart Buildings Using FRL

### 3.1. System Configuration

**Figure 2:**
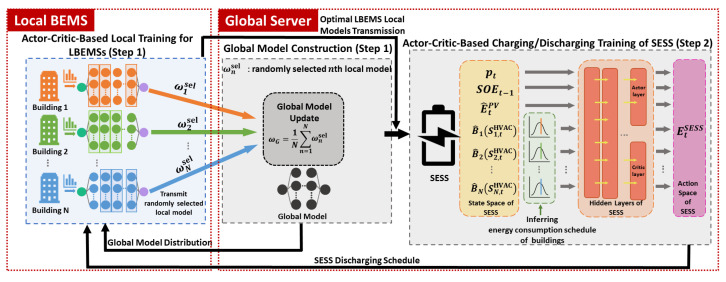
Training process for the proposed FRL-based energy management of smart buildings connected to a SESS.

We assume a situation in which the LBEMS manages the economic operation of HVACs, a major appliance of smart buildings, with a PV system. Multiple smart buildings are connected to a single SESS to reduce the total electricity cost of purchasing conventional energy from the grid. The charging and discharging operations of the SESS are controlled by the GS. In this study, we present an FRL-based BEMS that consists of a single GS and *N* LBEMSs, which is shown in Figure 2. Through the DRL process, under a TOU tariff, the HVAC agent in the LBEMS and the SESS agent in the GS conduct 24-h operation scheduling of their appliances with a 1-h scheduling resolution. As shown in Figure 2, the training process for the proposed approach includes the following two steps:

- Step (1) FRL for HVAC energy consumption scheduling: each HVAC agent in the LBEMS trains its own model to schedule the energy consumption of HVAC using the actor–critic method with its data. The randomly selected part of trained local models (i.e., the weights $\omega_{n}$ of its local neural network for LBEMS *n*) are periodically transmitted to the GS. Subsequently, the GS aggregates and updates the global model (i.e., the weights $\omega_{G}$ of the global neural network) using the federated stochastic gradient descent (FedSGD) algorithm [30] that averages the local models ($\omega_{G}=\frac{1}{N}\sum_{n=1}^{N}\omega_{n}$). The updated global model is distributed to all LBEMSs where all HVAC agents update their own models based on the global model. The updated local models and global model are exchanged iteratively until a predetermined stopping criterion is satisfied.
- Step (2) SESS charging/discharging: the optimal HVAC energy consumption models calculated from Step (1) along with the fixed loads in the building are fed back into the GS where the SESS agent trains the model for charging and discharging energy from and to the utility and the LBEMSs using the actor–critic method. The trained discharging schedules are transmitted to the LBEMSs, and these schedules are added to the HVAC energy consumption schedules that are calculated by the HVAC agents in Step (1).

Note that the GS could be hacked by potential adversaries, thereby leading to a more serious privacy threat to all LBEMSs. To resolve this, we adopted a selective parameter sharing model for the FRL process in Step (1). The primary feature of the selective parameter sharing method is that each LBEMS *n* chooses and delivers a part of its local neural network model ($\omega_{n}^{\mathrm{sel}}\subset \omega_{n}$) to the GS. This method can prevent attackers from obtaining crucial information such as energy consumption of HVAC from the shared global neural network model. The mathematical formulation and the methodology for the proposed BEMS approach are described in the two subsections: the definition of state/action spaces and the reward functions for the DRL HVAC agent in the LBEMS and the DRL SESS agent in the GS in Section 3.2 and the proposed FRL-based energy management of the SESS with smart buildings along with the DRL method in Section 3.3.

### 3.2. System Description for HVAC and SESS Agents

#### 3.2.1. State Space

∀t=1,…,24, the state spaces for the HVAC agent in the LBEMS *n* and the SESS agent in the GS, respectively, are defined as follows:

(4) $$\begin{array}{cc} S_{n,t}^{\mathrm{HVAC}} & =\{p_{t},{\hat{T}}_{t}^{\mathrm{out}},T_{n,t-1}^{\mathrm{in}},TC_{n,t},{\hat{E}}_{n,t}^{\mathrm{PV}}\} \end{array}$$

(5) $$\begin{array}{cc} S_{t}^{\mathrm{SESS}} & =\{p_{t},SOE_{t-1},{\hat{E}}_{t}^{\mathrm{PV}},\hat{B}\left(S^{\mathrm{HVAC}}\right)\}. \end{array}$$

In Equation (4), $S_{n,t}^{\mathrm{HVAC}}$ represents the state space of the HVAC agent for the LBEMS *n* at time *t*. The state $p_{t}$ is the price under the TOU rates at time *t*. ${\hat{T}}_{t}^{\mathrm{out}}$ is the predicted outdoor temperature at time *t*. $T_{n,t-1}^{\mathrm{in}}$ is the building *n*’s indoor temperature at time t−1. $TC_{n,t}=\frac{OCC_{n,t}}{Cap_{n}}$ is the thermal capacity of the building *n* at time *t* and it provides the population density in the building *n* where $OCC_{n,t}$ and $Cap_{n}$ represent the occupancy and thermal occupancy capacity of building *n*, respectively. ${\hat{E}}_{n,t}^{\mathrm{PV}}$ is predicted PV generation output of building *n* at time *t*. $T_{n,t}^{\mathrm{in}}$ is computed in terms of $T_{n,t-1}^{\mathrm{in}}$, ${\hat{T}}_{t}^{\mathrm{out}}$, $TC_{n,t}$, the energy consumption of HVAC in building *n* at time *t* ($E_{n,t}^{\mathrm{HVAC}}$), generated PV output for energy usage directly at time *t* (${\hat{E}}_{n,t}^{\mathrm{PV}}$), and the environmental parameters ($\alpha_{n},\beta_{n},\gamma_{n}$) that characterize the indoor thermal and occupancy condition using the following equation: $T_{n,t}^{\mathrm{in}}=T_{n,t-1}^{\mathrm{in}}+\alpha_{n}\left({\hat{T}}_{t-1}^{\mathrm{out}}-T_{n,t-1}^{\mathrm{in}}\right)+\beta_{n}\left(E_{n,t}^{\mathrm{HVAC}}+{\hat{E}}_{n,t}^{\mathrm{PV}}\right)$+$\gamma_{n}TC_{n,t}$. $\alpha_{n}$, $\beta_{n}$, and $\gamma_{n}$ are the parameters that identify the impact of temperature, energy consumption for the HVAC, and the building’s thermal occupancy density, respectively, on the indoor temperature of the building. We consider a situation in which the energy generated from the PV system is preferentially used for HVAC energy consumption in the building. In Equation (5), $S_{t}^{\mathrm{SESS}}$ represents the state space of the SESS agent in the GS at time *t*. The state $SOE_{t-1}$ is the state of energy (SOE) of the SESS at time t−1. The state ${\hat{E}}_{t}^{\mathrm{PV}}$ is the predicted PV generation output of SESS at time *t*. $\hat{B}\left(S_{t}^{\mathrm{HVAC}}\right)$ is the N×1 vector for the inferred energy data from fully trained neural network model for LBEMS energy usage including the fixed loads in terms of the state $S_{t}^{\mathrm{HVAC}}$, where $\hat{B}\left(S_{t}^{\mathrm{HVAC}}\right)=\left[{\hat{B}}_{1}\left(S_{1,t}^{\mathrm{HVAC}}\right),\ldots ,{\hat{B}}_{N}\left(S_{N,t}^{\mathrm{HVAC}}\right)\right]$ and ${\hat{B}}_{n}\left(\cdot \right)$ is the fully trained neural network of LBEMS *n* during the FRL process. For preserving the privacy of LBEMSs’s energy consumption data, the SESS does not receive any exact energy consumption data from the LBEMSs. The SESS receives fully trained neural network from each LBEMS and infers the energy consumption schedules of all LBEMSs using $\hat{B}\left(\cdot \right)$. The utilization of the fully trained LBEMS model can preserve the data privacy of buildings without explicitly sharing building’ energy consumption pattern with the GS.

#### 3.2.2. Action Space

**Figure 3:**
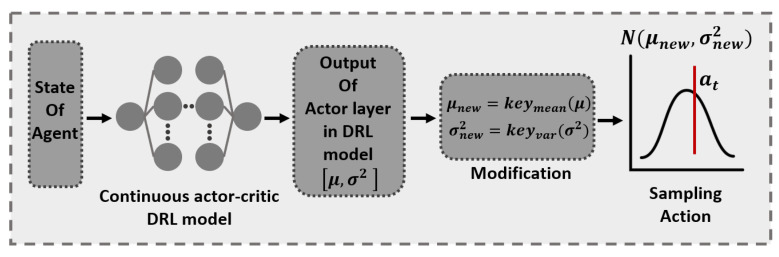
Sampling process of action for the proposed deep reinforcement learning (DRL) agent.

In this study, the action corresponds to the energy consumption schedule of the HVAC and the energy charging and discharging schedule of the SESS. The action is a key component for the HVAC and SESS operators because the energy scheduling of the HVAC and SESS directly affects the energy cost and comfort level of the buildings. Therefore, the optimal action for the HVAC and SESS should be determined by their DRL agents according to the environment and the state described in Equations (4) and (5). The action spaces of the HVAC and SESS are written as

(6) $$A_{n,t}^{\mathrm{HVAC}}=\left\{E_{n,t}^{\mathrm{HVAC}}\right\},\;A_{t}^{\mathrm{SESS}}=\left\{E_{t}^{\mathrm{SESS}}\right\}$$

where $E_{n,t}^{\mathrm{HVAC}}$ and $E_{t}^{\mathrm{SESS}}$ represent the energy consumptions of the HVAC for the LBEMS *n* and the charging/discharging energy of the SESS considering their own PV generation at time *t*, respectively. Given the predicted PV generation output, the operational dynamics of the SOE for the SESS at time *t* is expressed as follows: $SOE_{t}=SOE_{t-1}+E_{t}^{\mathrm{SESS}}+{\hat{E}}_{t}^{\mathrm{PV}}$. The HVAC agent and the SESS agent select continuous energy consumption and charging and discharging actions using normal distribution $N(\mu ,\sigma^{2})$ with the values of mean μ and variance $\sigma^{2}$ received from the actor network. Furthermore, to prevent the adversary from inferring the energy consumption data of buildings, the mean μ and variance $\sigma^{2}$ obtained from the actor network are recalculated by two key functions, $key_{mean}$ and $key_{var}$, which in turn yields new mean $\mu_{\mathrm{new}}$ and variance $\sigma_{\mathrm{new}}^{2}$ for action distribution as shown in Figure 3. Only the HVAC and SESS agents know these key functions; therefore, the adversaries can neither infer the exact energy consumption, nor the charging and discharging schedule from the neural network. In our proposed method, the SESS agent infers the energy consumption data of each building using neural network model and key functions of each LBEMS. Before the SESS agent selects the action, it observes the state that contains inferred LBEMSs’ energy consumption data. Then, the SESS agent selects the action (e.g., energy charging or discharging) based on the given state. The SESS agent also chooses its action based on normal distribution with the outputs received from its actor network.

#### 3.2.3. Reward Function

The reward functions for both HVAC and SESS agents are written as the sum of the negative energy cost and negative penalty cost, which are related to the building’s thermal comfort preference and the SESS operation. To begin with, the reward function for the HVAC agent *n* is expressed as

(7) $$\begin{array}{c} R_{n,t}^{\mathrm{HVAC}}=\left\{\begin{array}{c} -\left[p_{t}E_{n,t}^{\mathrm{HVAC}}+\bar{\epsilon_{n}}\left(T_{n}^{\min}-T_{n,t}^{\mathrm{in}}\right)\right],\;\mathrm{if}\;T_{n,t}^{\mathrm{in}}<T_{n}^{\min}\\ -\left[p_{t}E_{n,t}^{\mathrm{HVAC}}+\underline{\epsilon_{n}}\left(T_{t}^{\mathrm{in}}-T_{n}^{\max}\right)\right],\;\;\mathrm{if}\;T_{n,t}^{\mathrm{in}}>T_{n}^{\max}\\ -p_{t}E_{n,t}^{\mathrm{HVAC}},\;\;\;\;\;\;\;\;\;\;\;\;\;\;\;\;\;\;\;\;\;\;\;\;\;\;\mathrm{otherwise}, \end{array}\right. \end{array}$$

where $\bar{\epsilon_{n}}$ and $\underline{\epsilon_{n}}$ are the penalties for thermal discomfort in the building *n*. The discomfort cost is formulated as the deviation in the building *n*’s preferred temperature $T_{n,t}^{\mathrm{in}}$ from $T_{n}^{\min}$ and $T_{n}^{\max}$. This cost is regarded as a reward with a negative sign when $T_{n,t}^{\mathrm{in}}$ is out of the range of [$T_{n}^{\min}$, $T_{n}^{\max}$]. The reward function for the SESS agent is formulated as

(8) $$\begin{array}{c} R_{t}^{\mathrm{SESS}}=\left\{\begin{array}{c} -[p_{t}E_{t}^{\mathrm{SESS}}+\bar{\tau }\left(SOE_{t}-SOE^{\max}\right)],\\ \;\;\;\;\;\;\;\;\;\;\;\;\;\;\;\;\;\;\;\;\;\;\;\;\;\;\;\mathrm{if}\;SOE_{t}>SOE^{\max}\\ -[p_{t}E_{t}^{\mathrm{SESS}}+\underline{\tau }\left(SOE^{\min}-SOE_{t}\right)],\\ \;\;\;\;\;\;\;\;\;\;\;\;\;\;\;\;\;\;\;\;\;\;\;\;\;\;\;\mathrm{if}\;SOE_{t}<SOE^{\min}\\ -p_{t}E_{t}^{\mathrm{SESS}},\;\;\;\;\;\;\;\;\;\;\;\;\;\;\mathrm{otherwise}. \end{array}\right. \end{array}$$

Here, $\bar{\tau }$ and $\underline{\tau }$ are denoted by the penalties for the overcharging and undercharging of SESS, respectively. When the SOE is smaller than $SOE^{\min}$ (i.e., undercharging) or the SOE is greater than $SOE^{\max}$ (i.e., overcharging), energy underutilization of the SESS happens.

### 3.3. Proposed Privacy-Preserving Energy Management of the SESS with Smart Buildings

We describe Algorithm 1 for privacy-preserving energy management of the SESS with FRL-based distributed BEMS. The HVAC agents in the LBEMSs and the SESS agent in the GS employ the DRL algorithm based on actor–critic method that is illustrated in Section 2.2. In this algorithm, the LBEMS and GS first interact iteratively and train the energy consumption model for the HVAC agents in the LBEMSs during the FRL process until all HVAC agents find their own optimal consumption model (line 5∼20 in Algorithm 1). All HVAC agents obtain their optimal model using DRL method, and these agents are assumed to start their learning procedure synchronously. The optimal models trained by the HVAC agents during the FRL process are then delivered to the GS where the charging and discharging schedule of the SESS agent is trained using DRL method (line 23∼31 in Algorithm 1). Finally, the trained discharging schedule of the SESS agent is transmitted and added to the energy consumption schedule of the HVAC agents in the LBEMSs. The overall learning procedure followed by the proposed energy management of smart buildings in Algorithm 1 is illustrated as belows:

1. Prior to the learning procedure, the energy consumptions and discomfort parameters of both HVAC and SESS agent are initialized (line 1).
2. Probability of actions, weights of the actor network and the critic network, Q-value for the HVAC and the SESS agent are initialized (line 2).
3. The global neural network model $\omega_{G}$ in the GS along with the sharing batch φ for the FRL approach is initialized (line 3).
4. During every local training episode per communication round, each building’s HVAC agent iterates the following procedures to compute its optimal energy consumption schedule from t=1 to t=24 (line 6∼13).
  (a) Sample action $a_{n,t}^{\mathrm{HVAC}}$ based on distribution $N(\mu_{n,t}^{\mathrm{HVAC}},{\left\{\sigma_{n,t}^{\mathrm{HVAC}}\right\}}^{2})$ generated by actor network and key functions in state $s_{n,t}^{\mathrm{HVAC}}$ (line 8).
  (b) Execute action $a_{n,t}^{\mathrm{HVAC}}$, receive reward $R_{n,t+1}^{\mathrm{HVAC}}$ from the action and $V_{\pi_{\theta_{c}}^{\mathrm{HVAC}}}\left(s_{n,t}^{\mathrm{HVAC}}\right)$ from the critic network, and finally, calculate the target value of critic network, $TD_{t}$ (line 9).
  (c) Compute the loss functions of actor network and critic network to minimize the losses and update the model of the HVAC agent using the ADAM optimizer [39] (lines 10, 11).
5. The HVAC agent *n* randomly selects a part of its local model $\omega_{n}^{\mathrm{sel}}$ from the fully trained model $\omega_{n}$ and transmit it to the GS where it is stored in batch φ (lines 14, 15).
6. The GS yields a global neural network model $\omega_{G}^{\mathrm{new}}$ by executing the FedSGD method with the selected weights in φ (line 17).
7. This newly generated global model $\omega_{G}^{\mathrm{new}}$ is distributed to all HVAC agents in LBEMSs where those agents resume their own training based on $\omega_{G}^{\mathrm{new}}$ (lines 18, 19).
8. All HVAC agents transmit their fully trained model $\hat{B}\left(\cdot \right)$ to the GS (line 21).
9. For training episodes, the SESS agent repeats the following procedures to search for an optimal charging and discharging schedule from t=1 to t=24 (line 23∼31).
  (a) The SESS agent infers the energy consumption of the LBEMS *n* using the model ${\hat{B}}_{n}\left(\cdot \right)$ and the state $S_{n,t}^{\mathrm{HVAC}}$ (line 25).
  (b) Sample an action $a_{t}^{\mathrm{SESS}}$ based on distribution $N(\mu_{t}^{\mathrm{SESS}},{\left\{\sigma_{t}^{\mathrm{SESS}}\right\}}^{2})$ generated by the actor network and the key functions given by state $s_{t}^{\mathrm{SESS}}$, which includes the inferred energy consumption data for all LBEMSs (line 26).
  (c) Compute action $a_{t}^{\mathrm{SESS}}$, receive reward $R_{t+1}^{\mathrm{SESS}}$ and $V_{\pi_{\theta_{c}}^{\mathrm{SESS}}}\left(s_{t}^{\mathrm{SESS}}\right)$ from the critic network, and calculate $TD_{t}$ of the SESS agent (line 27).
  (d) Estimate the loss functions of the actor network and the critic network by minimizing them, and update the model of the SESS agent using the ADAM optimizer (line 28, 29).

**Algorithm 1:** FRL-based energy management of a SESS with multiple smart buildings.
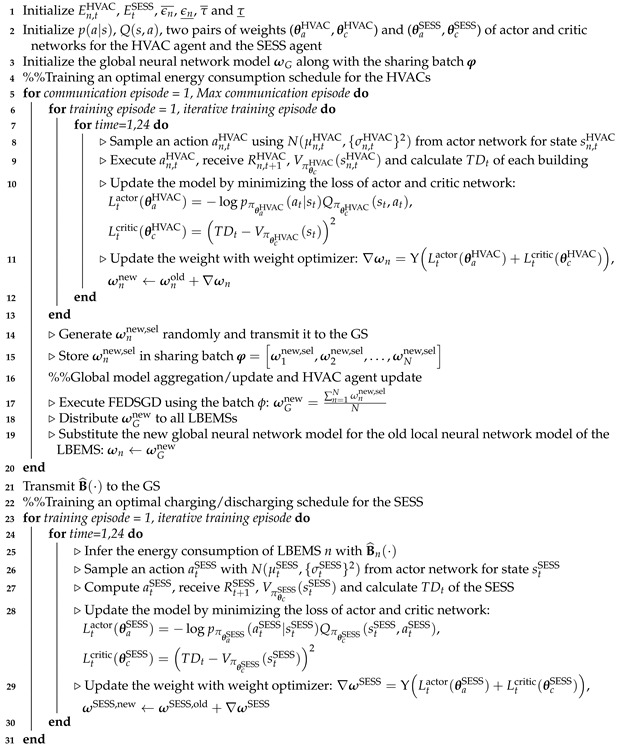


## 4. Simulation Results

### 4.1. Simulation Setup

**Figure 4:**
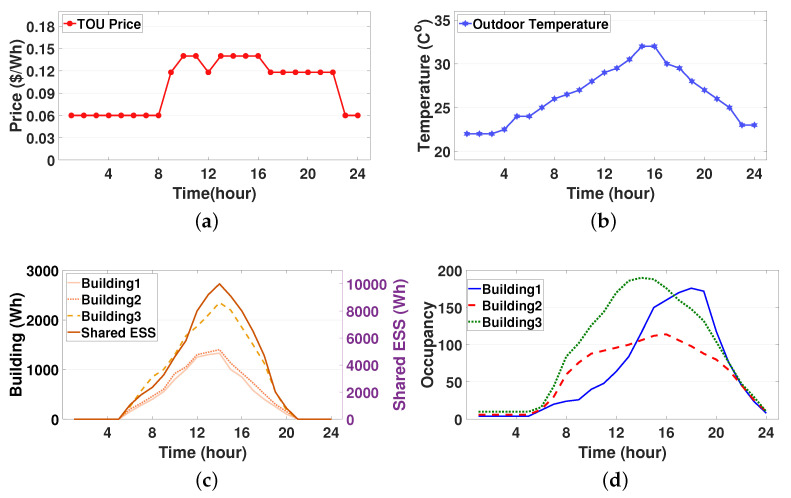
Parameters of electricity price, weather condition, and building occupancy: (**a**) time-of-use (TOU) price; (**b**) predicted outdoor temperature; (**c**) predicted photovoltaic (PV) generation output; and (**d**) daily occupancy.

Under the TOU pricing, the predicted outdoor temperature and the PV generation output are plotted in Figure 4a–c. Accordingly, we considered three smart buildings with PV systems that are connected to a single SESS with the PV system. Each building was equipped with one controllable HVAC system and had different environment in terms of consumer comfort, thermal characteristics, HVAC capacity, occupancy capacity, and building occupancy which are detailed in Table 1 and plotted in Figure 4d. For the SESS, the battery capacity was 280 kWh; moreover, the maximum charging and discharging energy were both 120 kWh. The minimum, maximum and initial SOE of the SESS were set to 10%, 100% and 50% of its capacity, respectively. The penalties for SESS overcharging and undercharging were $\bar{\tau }$ = 600 and $\underline{\tau }$ = 600, respectively. The actor–critic DRL models of the HVAC and SESS agents consist of: (i) four hidden layers for a common body network that has 512 neurons in the first layer and 256 neurons each from the second layer to the fourth layer and (ii) two hidden layers for each of the actor network and the critic network having 128 neurons. We adopted a hyperbolic tangent function as a transfer function for the HVAC and SESS DRL models. The ADAM algorithm was selected to train the HVAC and SESS agents with a learning rate of 0.00005 and 0.00004, respectively. Two key functions, $key_{mean}$ and $key_{var}$, for calculating new mean and variance of action distribution for the HVAC and SESS agents were selected as sigmoid function and exponential function, respectively. It is assumed that a single communication round between the LBEMS and the GS occured every 150 iterations that are required for the LBEMS training. The test was executed using Python 3.7.0 and Pytorch 1.1.0.

### 4.2. Performance Assessment

#### 4.2.1. Training Curve Convergence

**Figure 5:**
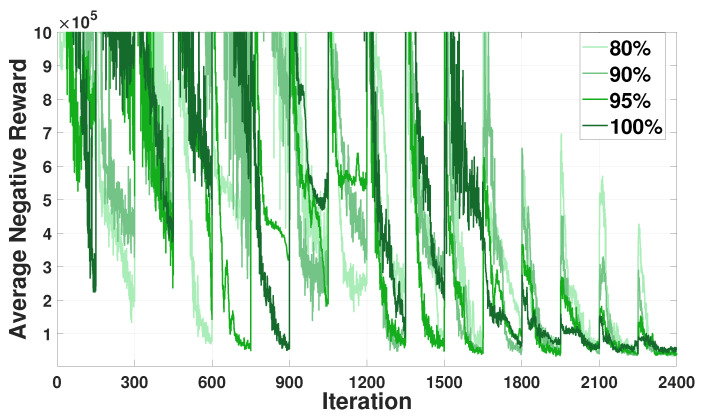
Average negative reward convergence for the heating, ventilation, and air conditioning (HVAC) agents with varying amounts of selective parameter sharing.

Figure 5 depicts the training curves for average negative rewards for the HVAC agents in the three buildings under varying amounts of selected parameter sharing. We first observed that the training curves decreased and converged well during the learning process, while a sharp fluctuation occurred in every communication round (150 iterations). Another observation was that a smaller sharing of the selected parameters yielded a worse convergence performance. These results indicate a tradeoff relationship between the fast convergence of the training curve and the privacy preservation through sharing a lesser amount of selective parameters. In the subsequent simulation study, we considered that the HVAC agents share 80% of their DRL model with the GS to preserve energy usage information of the building occupants.

**Figure 6:**
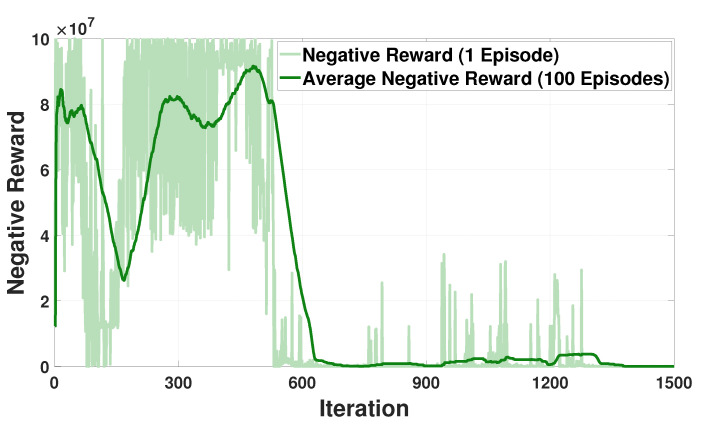
Negative reward convergence for the SESS agent.

Figure 6 illustrates the training curves for negative reward (per one episode) and average negative reward (per 100 episodes) for the SESS agent. As mentioned in Section 3.2, the SESS agent utilizes fully trained neural networks of all LBEMSs’ agents for preserving the privacy of buildings when learning the optimal policy of energy charging and discharging. We verified from Figure 6 that the training curves of the SESS agent converges well although the SESS agent uses neural network models rather than exact energy consumption data of LBEMSs.

**Figure 7:**
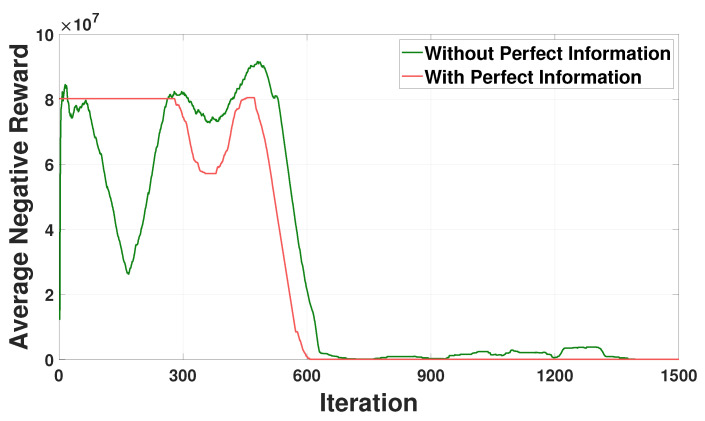
Comparison of average negative reward convergence for the SESS agent between with and without perfect energy consumption information of HVACs.

Figure 7 compares the training curves for average negative rewards for the SESS agent between two schemes with perfect and without perfect energy consumption information of HVACs. In this figure, the scheme without the perfect energy consumption information of HVACs represents our proposed model where the neural network model of the HVAC agent is delivered to the GS and used as the element of the state space of the SESS agent. The SESS agent then infers the energy consumption schedules of HVACs using their neural network models. By contrast, the scheme with the perfect energy consumption information of HVACs considers the situation where the HVAC energy consumption schedule (i.e., the action of the HVAC agent in (6)) calculated by the HVAC agent is used as the element of the state space of the SESS agent instead of the neural network model of the HVAC agent. Note from Figure 7 that both the training curves without and with the perfect energy consumption information converge well, however the former converges rather slower than the latter. This is because inferring the energy consumption schedules of all HVACs using their neural network models requires additional training iterations of the SESS agent. We conclude from the result of Figure 7 that the proposed approach can preserve the privacy of HVAC energy consumption data at the expense of the convergence speed of the training curve.

#### 4.2.2. HVAC Energy Management

**Figure 8:**
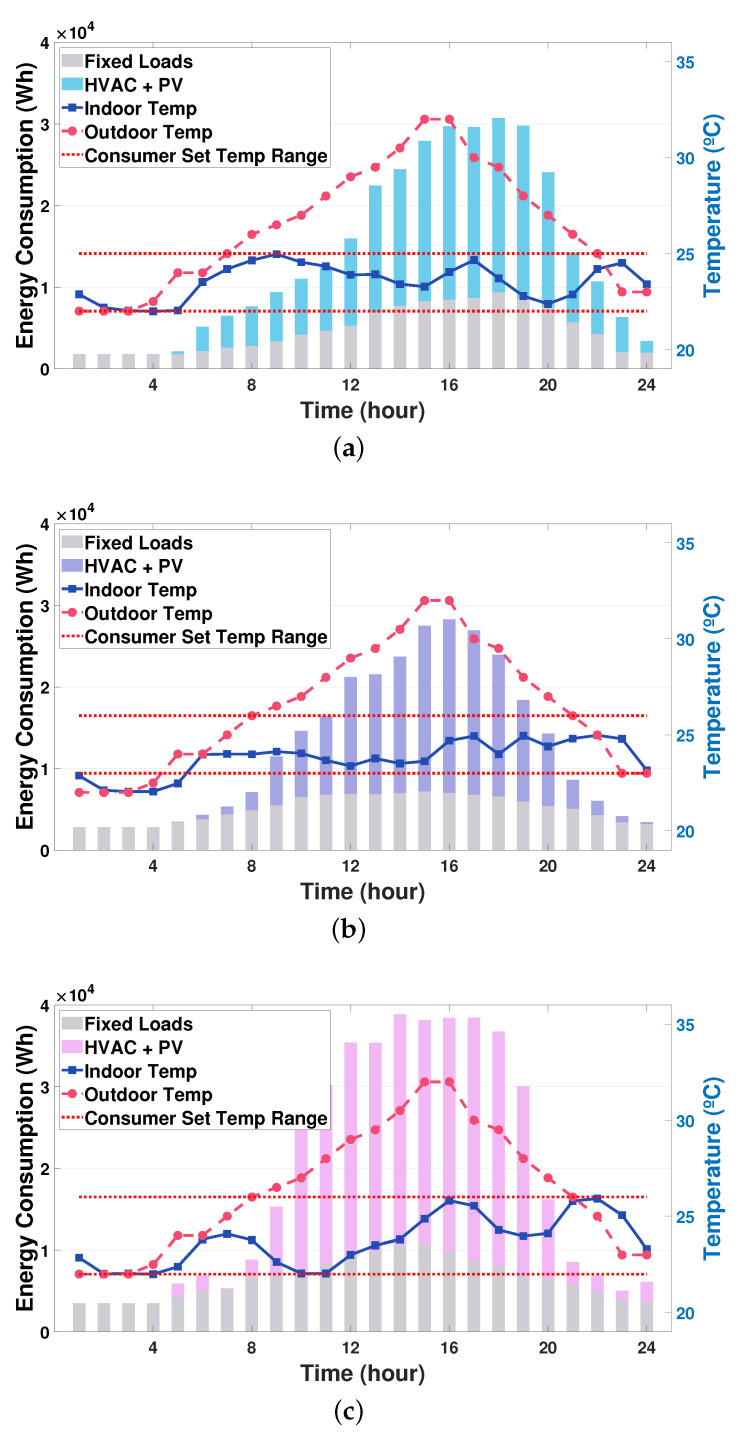
Sum of scheduled energy consumption for HVAC ($E_{n,t}^{\mathrm{HVAC}}$+${\hat{E}}_{n,t}^{\mathrm{PV}}$) and the fixed loads: (**a**) building1; (**b**) building2; and (**c**) building3.

The graphs in Figure 8 show the aggregated scheduled optimal energy consumption (action $E_{n,t}^{\mathrm{HVAC}}$) of the HVAC system considering predicted PV generation and the fixed loads for the three buildings. Specifically, in individual building, the optimal energy consumption schedule for the HVAC was computed after the training curves converge as shown in Figure 5. It can be observed from these graphs that the optimal energy consumption schedule of the HVAC in each building varies according to its heterogeneous building environment, such as the preferred indoor temperature, thermal characteristics, and the building occupancy. In Figure 8a, building1 consumes a large amount of the HVAC energy in the time period (5:00 p.m. to 8:00 p.m.) even though the outdoor temperature decreases. This is because building1 has a high occupancy with a large value of γ during this time period as shown in Figure 4d and Table 1. By contrast, it is observed from Figure 8b that building2 consumes more (or less) HVAC energy as the outdoor temperature increases (or decreases). This is because larger α and β in building2 makes the HVAC energy consumption rely increasingly on the outdoor temperature. Furthermore, because the outdoor temperature during the time period (1:00 a.m. to 4:00 a.m.) is lower than the preferred minimum indoor temperature of building2, the indoor temperature decreases gradually even though the HVAC turns off during this time period. Moreover, it should be noted that among the three buildings considered, building3 consumes the largest HVAC energy for most of the time periods. This derives from the fact that building3 has the largest building occupancy and β that represents the sensitivity of current indoor temperature to the HVAC energy consumption.

#### 4.2.3. SESS Charging and Discharging Management

**Figure 9:**
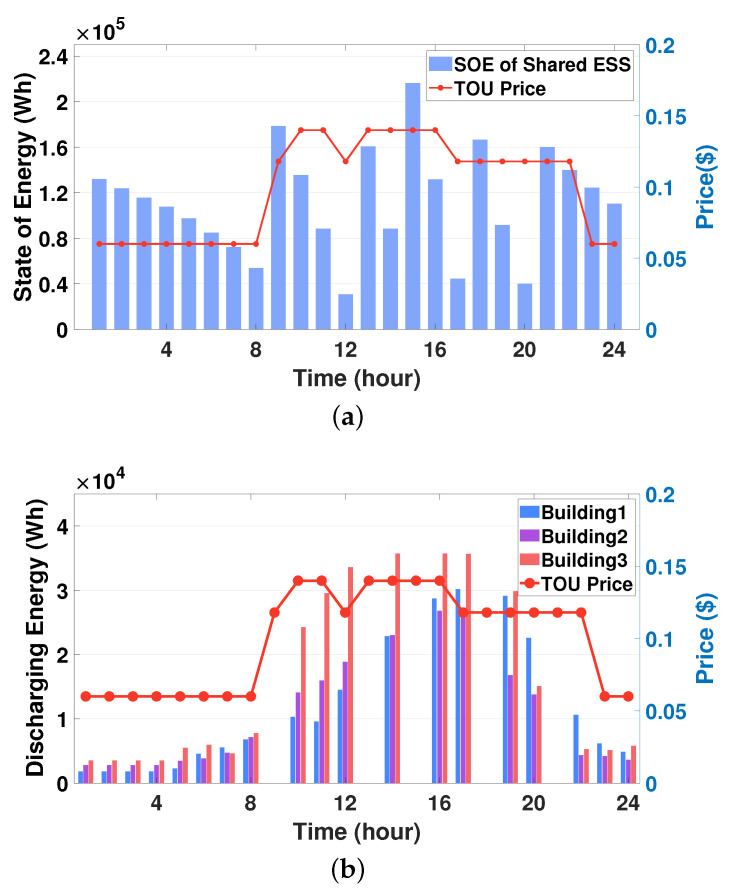
Simulation results for the SESS: (**a**) state of energy (SOE) (state $SOE_{t}$) of the SESS; (**b**) discharging energy schedules (action $E_{t}^{\mathrm{SESS}}$) for the three buildings.

**Figure 10:**
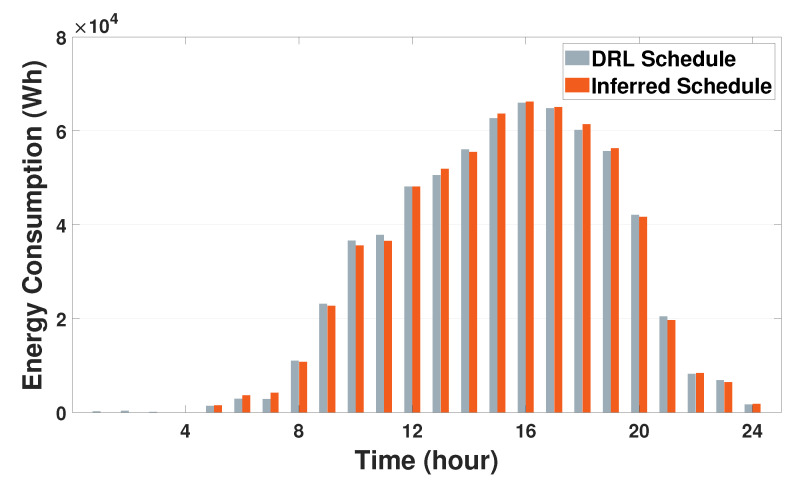
Comparison of the sum of energy consumption schedules for three buildings using the energy consumption schedule ($E_{n,t}^{\mathrm{HVAC}}$) of DRL HVAC agents and using the inferred energy consumption schedule (${\hat{B}}_{n}\left(S_{n,t}^{\mathrm{HVAC}}\right)$) of DRL SESS agent.

Figure 9a,b depict the SOE schedules of the SESS and the discharging energy schedules from the SESS to the three buildings, respectively. From Figure 9a, it can be observed that a sudden increase of the SOE occurs at five different times (9:00 a.m., 1:00 p.m., 3:00 p.m., 6:00 p.m., and 9:00 p.m.). At these times, the SESS charges energy from the grid (i.e., the SESS purchases energy from the grid) to supply building loads. At other times, it discharges energy, thus resulting in decreased SOE to satisfy the energy demand for the three buildings. The discharging energy schedules for these three buildings are plotted in Figure 9b where no bar graphs are shown for the charging times mentioned above. Note from Figure 9b that building3 receives more discharging energy from the SESS than buildings 1 and 2 during daytime. This is because the aggregated energy consumption schedule for building3 is greater than that for the other buildings, as illustrated in Figure 8. Another observation is that the SESS discharges more energy to building1 than building2 during the time period (4:00 p.m. to 10:00 p.m.). This is due to the fact that building1 has larger occupancy and aggregated energy demand than building2 during this time period. From Figure 9a,b, it can be concluded that the proposed DRL-based SESS control method calculates a moderate charging and discharging schedule of the SESS for multiple buildings with heterogeneous environments. In addition, to preserve the privacy for buildings’ energy consumption data, the SESS agent infers the corresponding schedules of all buildings based on the LBEMS energy consumption model $\hat{B}\left(\cdot \right)$ in Section 3.2 without using the optimal building energy consumption schedule calculated by the HVAC agent. Figure 10 shows the sum of energy consumption schedules for three buildings during a day, which is calculated by DRL HVAC agents and DRL SESS agent, respectively. Note that the sum of DRL SESS agent-based energy consumption schedules is inferred using the LBEMS energy consumption model $\hat{B}\left(\cdot \right)$. We verify from this figure that the energy consumption schedule inferred by the SESS agent using the LBEMS model deviates only 0.2∼1% from the optimal energy consumption schedule computed by the HVAC agent. In sum, the aforementioned results demonstrate that the SESS agent successfully calculates optimal charging or discharging action while preserving the privacy of buildings’ energy consumption.

#### 4.2.4. Flexibility with Varying Number of the HVAC Agents

**Figure 11:**
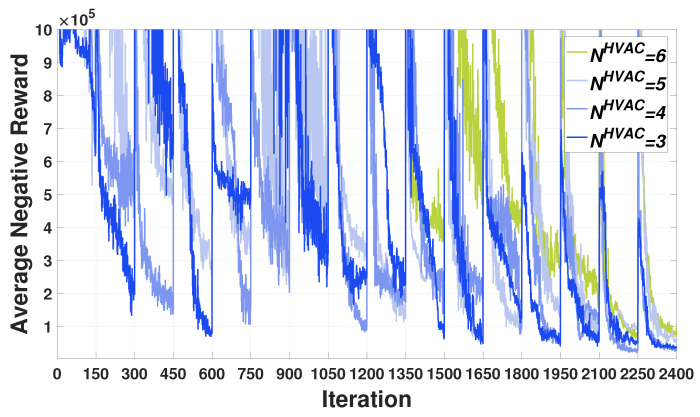
Average negative reward convergence with different number of HVAC agents ($N^{\mathrm{HVAC}}$).

Figure 11 compares the training curves for average negative rewards for the HVAC agents in terms of the number of the HVAC agents ($N^{\mathrm{HVAC}}$). We first observe from this figure that all training curves converge well with increasing number of the HVAC agents; however, the rate of convergence is rather slowing down as the number of the HVAC agents increases. Next, in the plot for $N^{\mathrm{HVAC}}$=6, we considered the situation in which an additional building (sixth HVAC agent) with the HVAC joined the FRL network that has the five HVAC agents at the 10th communication round (1350 iterations). In this situation, we observe from Figure 11 that after the 10th communication round, the training curve with $N^{\mathrm{HVAC}}$=6 starts increasing more significantly than the training curves with $N^{\mathrm{HVAC}}$=1∼5. This observation is because the new HVAC agent joins the FRL network. After the 15th communication round (2100 iterations), we verify that the training curve with $N^{\mathrm{HVAC}}$=6 converges to its optimal policy. We conclude in Figure 11 that our FRL framework is flexible to a varying number of agents including the agent joining scenario.

#### 4.2.5. Performance Comparison between the Proposed Approach and Existing Methods

**Figure 12:**
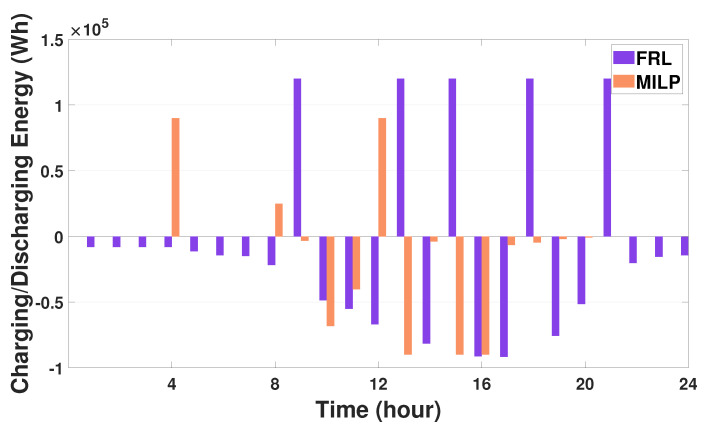
Comparison of hourly charging and discharging energy ($E_{t}^{\mathrm{SESS}}$) of SESS between the proposed FRL method and the mixed-integer linear programming (MILP) method.

We compared the performance of our proposed FRL method to that of two existing methods that employ the distributed multi-agent model without FRL and the mixed-integer linear programming (MILP) optimization model, respectively. The simulation result showed that the electricity cost of HVAC systems using the proposed approach deviates only 1.1∼1.4% from the electricity cost of HVAC systems using the distributed multi-agent model without FRL. In addition, we verified from the simulation study that compared to the MILP method, our proposed method enables all three HVAC agents to reduce 24∼32% of energy consumption and 18.6∼20.6% of electricity cost. Figure 12 compares the charging or discharging energy of the SESS between the MILP method and the proposed FRL method. In this figure, a positive (or negative) value represents the amount of charging (or discharging) energy. It can be verified from Figure 12 that the proposed FRL method discharges more energy than the MILP method during a day; the SESS agent using the proposed method can support 66% of the total energy consumption of the buildings whereas the MILP method can support 35% of the total energy consumption of the buildings. Therefore, these results indicated that building energy management integrated with the DRL-based HVAC and SESS agents leads to a more economic energy consumption scheduling compared to the conventional optimization approach.

#### 4.2.6. Computational Efficiency

We evaluated the total computation time for the proposed algorithm, which is defined as the sum of the maximum training time among the three HVAC agents and the training time for the SESS agent. The proposed method requires a total of 512 s (368 s and 144 s for the HVAC and SESS agent trainings, respectively) to find their optimal policy. The proposed algorithm is carried out for one day with a 1-h scheduling resolution; it is hence computationally efficient. In addition, we quantified the total computation time for the distributed multi-agent method without FRL. This method requires a total of 430 s (292 s and 138 s for the HVAC and SESS agent trainings, respectively). Compared to the distributed multi-agent method without FRL, the proposed approach requires only additional 82 s.

## 5. Discussions

### 5.1. Various Types of Controllable Appliances in the Smart Building

In this study, we considered that each LBEMS calculates the energy consumption schedule of only HVAC appliance that consumes the largest energy consumption in the smart building. However, it is possible that various types of building appliances such as refrigerators, elevators and electric vehicles can be controlled by the LBEMS to reduce the electricity cost of the building [40,41]. In the previous studies, the controllable building appliances can be managed by the LBEMSs to find their economic energy consumption schedule according to their operation characteristics. To include these controllable appliances in our proposed framework, a key part would be to design state and action spaces and reward functions for the DRL agents of these appliances based on their unique operation characteristics. Thus, the DRL-based LBEMS model with additional appliance agents can be merged into the FRL-based framework proposed in Section 3 to schedule the optimal energy consumption of multiple smart buildings while preserving the privacy of the energy consumption of each smart building.

### 5.2. Practical Model of Building Thermal Dynamics

For simplicity, we approximated a real-world HVAC system model by focusing on analyzing the impact of the outdoor temperature, the building occupancy, and the HVAC energy consumption on the indoor temperature of the building. In addition, the DRL-based LBEMS was implemented in a single-zone building model rather than a multizone building that has multiple temperature zones. However, we emphasize that our study is the first step toward constructing the privacy-preserving FRL framework to schedule the energy consumption of smart buildings connected to the SESS. An important extension of our study is to construct the FRL-based energy management framework for multizone buildings with a complete model of the HVAC including the operation and air temperature of supply fan and external thermal disturbance [8,42], and it is referred to as a future work.

## 6. Conclusions

This paper presented a distributed and privacy-preserving FRL algorithm that conducts the energy management of a smart community that comprised of one SESS and multiple smart buildings equipped with the HVACs. The presented framework has the following two key components: (i) an FRL module that enables the HVAC agent and the GS to collaboratively learn the optimal consumption model of the HVAC while preserving the privacy of buildings’ energy consumption data using a selective parameter sharing method, and (ii) an SESS management module that enables the SESS agent to train the optimal charging and discharging model of the SESS without having the knowledge of the optimal building energy consumption schedule. Simulation results are presented for a smart community comprised of one SESS and three smart buildings with PV systems. The results demonstrated the effectiveness of the proposed approach in terms of training convergence, energy consumption schedule of the HVAC, charging/discharging schedule of the SESS, flexibility under different number of the HVAC agents, decreased energy consumption as compared to the conventional optimization approach, and computation time.

## Figures and Tables

**Table 1:** Simulation parameters in the three buildings.

Parameter	Building1	Building2	Building3
$T^{\min}$	23 °C	23 °C	22 °C
$T^{\max}$	25 °C	26 °C	26 °C
$\bar{\epsilon }$, $\underline{\epsilon }$	13,000	16,000	21,000
α	0.85	0.92	0.88
β	−0.0004	−0.000325	−0.00022
γ	1.25	0.8	0.75
Cap	125	130	180
$E^{\mathrm{HVAC},\max}$	22 kWh	24 kWh	30 kWh

## Data Availability

Not applicable.
